# Synergetic Effect and Phase Engineering by Formation of Ti_3_C_2_T_x_ Modified 2H/1T-MoSe_2_ Composites for Enhanced HER

**DOI:** 10.3390/ma16216991

**Published:** 2023-10-31

**Authors:** Lei Xiao, Qichao Yang, Xiangyang Zhu, Yang Wei, Jing Wang

**Affiliations:** 1 School of Integrated Circuits, Tsinghua University, Beijing 100084, China; xiaolei@mail.tsinghua.edu.cn; 2 School of Materials Science and Engineering, University of Science and Technology Beijing, Beijing 100083, China; 18866986789@163.com (Q.Y.); 202310192378@mail.scut.edu.cn (X.Z.); 3 Department of Physics and Tsinghua-Foxconn Nanotechnology Research Center, Tsinghua University, Beijing 100084, China

**Keywords:** MoSe_2_@Ti_3_C_2_T_x_ heterostructures, hydrogen evolution reaction, phase structure

## Abstract

The typical semi conductivity and few active sites of hydrogen evolution of 2H MoSe_2_ severely restrict its electrocatalytic hydrogen evolution performance. At the same time, the 1T MoSe_2_ has metal conductivity and plentiful hydrogen evolution sites, making it feasible to optimize the electrocatalytic hydrogen evolution behavior of MoSe_2_ using phase engineering. In this study, we, through a simple one-step hydrothermal method, composed 1T/2H MoSe_2_, and then used newly emerging transition metal carbides with several atomic-layer thicknesses Ti_3_C_2_T_x_ to improve the conductivity of a MoSe_2-_based electrocatalyst. Finally, MoSe_2_@Ti_3_C_2_T_x_ was successfully synthesized, according to the control of the additional amount of Ti_3_C_2_T_x_, to form a proper MoSe_2_/ Ti_3_C_2_T_x_ heterostructure with a better electrochemical HER performance. As obtained MoSe_2_@4 mg-Ti_3_C_2_T_x_ achieved a low overpotential, a small Tafel slope and this work offers additional insight into broadened MoSe_2_ and MXenes-based catalyst’s electrochemical application.

## 1. Introduction

Hydrogen energy is one of the most prevalent potential candidates for renewable clean energy with high energy capacity to replace fossil fuel [1,2,3]. However, up to now, the most used industrial hydrogen production is still electrochemical water splitting [4,5,6]. Electrochemical HER is a cathode double-electron-transfer reaction that begins with the Volmer step, in which electrons are transferred to the cathode surface, bind to H+ at an unoccupied active site, and produce adsorbed H*. Currently, noble metals are the most feasible catalysts for electrochemical water splitting to reduce overpotential and simultaneously facilitate reaction kinetics of hydrogen evolution reaction (HER) [7,8,9]. Their scarcity and high price significantly restrict further commercial development and large-scale applications of these noble metals for water-splitting. Hence, finding cheap and earth-abundant electrocatalytic electrodes is essential to accelerating the commercial application of hydrogen energy.

Two-dimensional transition-metal dichalcogenides (2D TMDs) have many promoting properties for HER; 2D TMD’s atomically thin-based structure increased reaction surface area and available active site [10], and different transition metals have various d orbitals. Thus, exhibiting different catalytic performance makes it feasible to improve the HER performance by doping different metal elements [11,12]. However, excellent catalytic activity and low cost compared to noble metals give it considerable potential in HER. MoSe_2_, as a member of the 2D transition-metal dichalcogenide (TMDC), has received widespread attention due to its corrosion stability [13], chemically tunable electronic properties [14,15,16,17,18]. MoSe_2_ can present as a octahedral coordination 1T (tetragonal symmetry, one layer per repeat unit, octahedral coordination) phase with metallic properties and a triangular coordination 2H (hexagonal symmetry, two lavers per repeat unit, trigonal prismatic coordination) phase with semiconductor properties. However, MoSe_2_ electrochemical HER performances mainly depend upon its nanostructure and Crystalline phase, but for 2H phase MoSe_2_, lack of active sites and weak conductivity restrict its performance in electrochemical HER [19,20,21]. Researchers use commonly used regulatory methods to improve their electrochemical performance such as strain engineering [22], surface/interface engineering [23], alloy engineering [24,25], defect engineering [26], and compounds with other materials [27,28,29,30]. However, designing and fabricating excellent MoSe_2_-based composite catalysts for HER is of great fundamental importance to promoting TMDC-catalyst use of clean energy.

In this study, we, through a simple one-step hydrothermal method, use an emerging type of 2D transition-metal carbide Ti_3_C_2_T_x_ (T = -F, -OH, -O terminal group) to improve the conductivity of MoSe_2_-based electrocatalysts, Ti_3_C_2_T_x_. A typical MXene material is a typically a layered material with a sandwich structure consisting of three alternating layers of Ti atoms and two alternating layers of C atoms. In addition, due to the introduction of a small number of residual chemical elements during the preparation process of acid-solution etching, the surface adsorbs rich terminal groups such as -F, -OH, -O, etc. As a conductor can improve the HER performance of MoSe_2_ [31], the addition amount of Ti_3_C_2_T_x_ must be controlled accordingly to form a proper MoSe_2_/Ti_3_C_2_T_x_ heterostructure which has a better electrochemical HER performance. A small Tafel slope (79 mV dec^−1^) and low overpotential (−185.29 mV) were achieved with the obtained MoSe_2_@4 mg-Ti_3_C_2_T_x_. However, the Ti_3_C_2_T_x_ improves the performance of 1H/2H nanoflower-like MoSe_2_ in HER and is still not researched. This work utilizes Ti_3_C_2_T_x_ as a composite substrate, and controls the MoSe_2_ two-phase ratio. Both of these simultaneously enhance catalytic activity, which has significance for applying TMDC materials in the HER catalytic field.

## 2. Experiment Section

### 2.1. Synthesis of Ti_3_C_2_T_x_ MXene Nanosheets

Firstly, via a relatively safe method, we combined a 5 wt % LiF powder and a 1 mol/HCl solution-etching in a glove box inert atmosphere, using a magnetic force-driving stirred reactor for the reaction. After heating in an oil bath for 96 h, AlF_3_ is generated from the bulk-layered MXene phase, and Ti_3_AlC_2_ raw material is used to remove Al and generate Ti_3_C_2_T_x_. Then, ultrasonic dispersion and cleaning are carried out to obtain the required few-layer Ti_3_C_2_T_x_ thin sheets. The material was then dried in a vacuum-drying oven for 12 h. The product was then ground into powder in an agate mortar, collected, and vacuumed, successfully preparing layered Ti_3_C_2_T_x_.

### 2.2. Carbon Cloth Preprocess for Hydrophilic Treatment

According to the volume ratio of 4:1, we mixed 10% HNO_3_ and 20% H_2_SO_4_; soaked the carbon cloth (1 cm × 1 cm) at room temperature for 12 h; took it out, and put it in ultrasonic ethanol solution for 30 min; then, used DI water wash for 30 s; and repeated 3 times to remove the residual acid. It must be sealed and stored in deionized water.

### 2.3. Preparation of 1T/2H Phase MoSe_2_@Ti_3_C_2_T_x_ on CC

**Figure 1:**
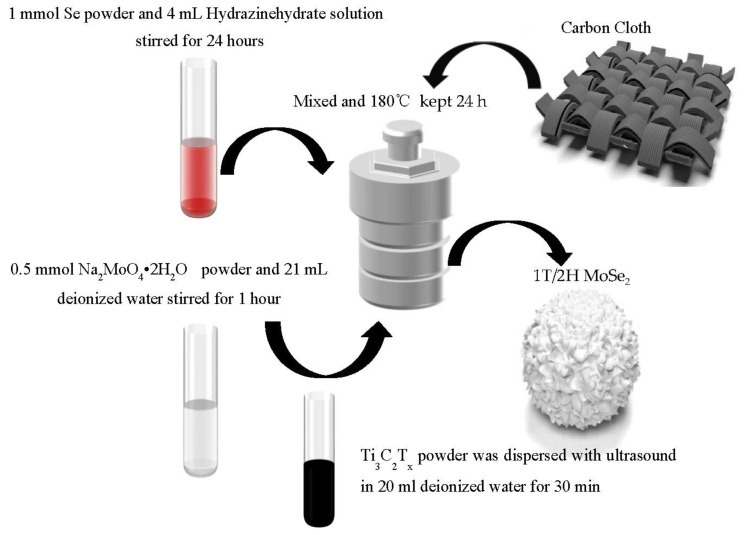
Schematic diagram of hydrothermal preparation of 1T/2H MoSe_2_@Ti_3_C_2_T_x_.

Figure 1 shows that Se powder (1.0 mmol) was dissolved into 15% hydrazine hydrate. Magnetic solution was stirred and kept for 24 h. At the same time, a few Ti_3_C_2_T_x_ powders were dispersed with ultrasound (600 W) in 20 mL deionized water for 30 min; then, 1 mmol of Na_2_MoO_4_ · 2H_2_O was added to the dispersion and stirred for 45 min. After that, we added the reduced Se ion solution to the dispersion and sealed it in a 50 mL high-pressure reactor, using a carbon cloth (1 cm × 1 cm) as the loaded substrate, and then heated it to 180 °C for insulation for 24 h. After cooling, the sample was washed and dried in a vacuum environment of 60 °C. During the process, attention should be paid to preventing oxidation.

### 2.4. Characterization

The morphology of the sample was characterized using field emission scanning electron microscopy (FEI, HONG KONG, SEM, FEI Nova 400), and X-ray diffraction (XRD) patterns were recorded using Cu Ka radiation on Rigaku D/Max 4000 V to determine sample-composition information. Raman spectral information was obtained using a confocal microscope (HORIBA FRANCE LabRAM HR Evolution), using a 532 nm laser at low power. XPS (Thermo ESCALAB 250XI) determined the chemical configuration.

### 2.5. Electrochemical Measurement

The electrocatalytic HER measurement of the obtained products was conducted using a standard three-electrode electrochemical workstation consisting of an electrode counter electrode (Pt), a reference electrode Ag/AgCl, and a stainless-steel working electrode. All the electrocatalytic HER measurements were performed in 1 mol/L H^+^ solution. Electrode potential was converted into reversible hydrogen electrode potential (vs. RHE), and solution IR compensation was performed. The LSV curves of the prepared material were measured at different scanning rates in an inert constant-room-temperature environment to characterize its catalytic hydrogen evolution performance. Electrochemical impedance spectroscopy and scanning CV curves were also measured to obtain the electrochemical surface area.

## 3. Result and Discussion

### Characterization of Ti_3_C_2_T_x_

**Figure 2:**
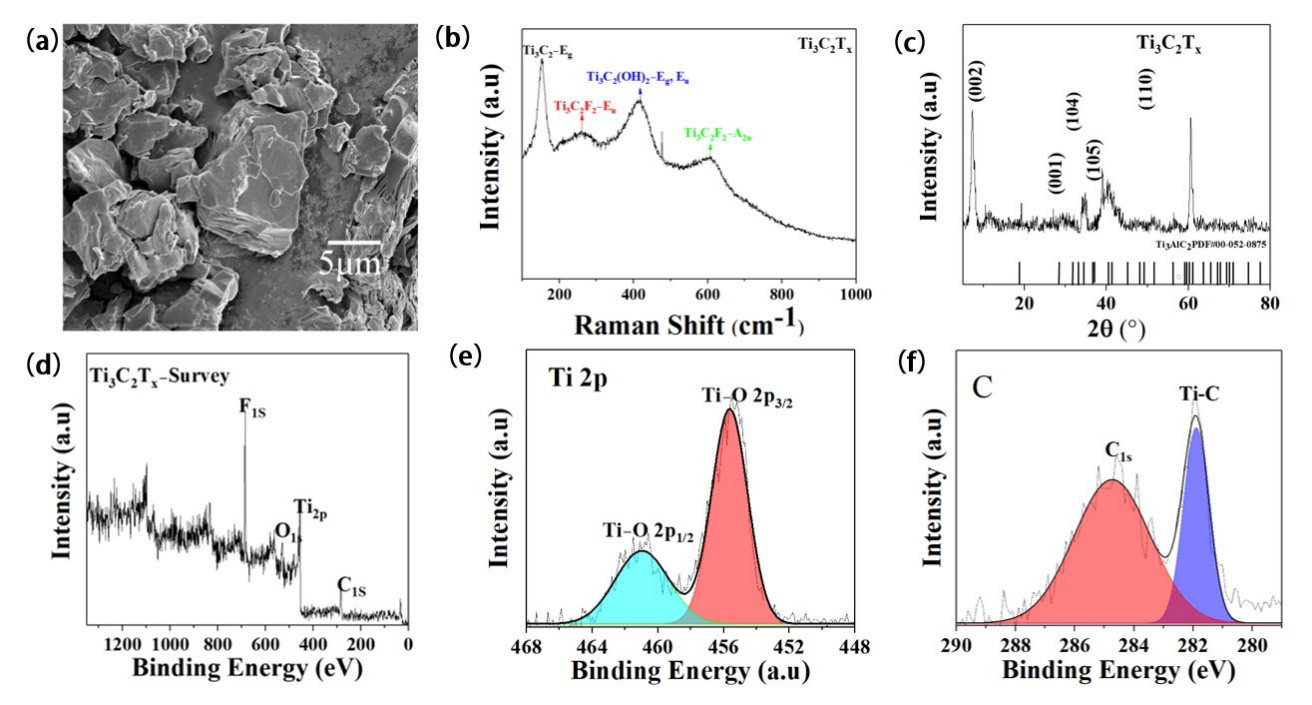
(**a**) SEM image; (**b**) Raman Spectrum; (**c**) XRD; and (**d**–**f**) XPS of Ti_3_C_2_T_x_.

First, the Ti_3_C_2_T_x_ was characterized using a Scanning Electron Microscope shown in Figure 2a Ti_3_C_2_T_x_ which has prominent layered characteristics, which allows for a large contact area with MoSe_2_ and CC. However, due to introducing some terminal groups on the surface of Ti_3_C_2_ during the preparation process, the Raman spectrum was used to characterize its molecular vibration. As shown in Figure 2b, in addition to its inherent interlayer vibration mode E_g_ peak, there were three apparent peaks at 235 cm^−1^, 373 cm^−1^, and 610 cm^−1^, which, respectively, represent the vibration modes of terminal groups such as F and OH, which are the same as previously reported [32]. To determine the phase-composition structure of the Ti_3_C_2_T_x_, we carried out XRD, as shown in Figure 2c. Ti_3_C_2_T_x_ had prominent peaks near 7.3°, 34.9°, 35.2° and 63°, which, respectively, correspond to the (002), (001), (104), (105) crystal planes of Ti_3_C_2_ [33], and the sharp peak represents its good crystallinity. However, XPS was used further to determine the element composition and valence state of Ti_3_C_2_T_x_. As shown in Figure 2d, the XPS full spectra of Ti_3_C_2_T_x_ showed distinctive peaks of Ti and OH F, O. The Ti 2p XPS spectra after deconvoluting is shown in Figure 2e, where there are double peaks at lower binding energies of 455.6 eV and higher binding energies of 465 eV were obtained, confirming the existence of oxygen-containing terminal groups, which further confirmed our previous Raman results. The C XPS spectra after deconvoluting are shown in Figure 2f; double peaks at lower binding energies of 284.8 eV and higher binding energies of 281.8 eV were obtained after Gaussian Lorentz fitting. The stoichiometric ratio of Ti and C is about 100:68. However, The Ti_3_C_2_T_x_ we obtained was rich in oxygen, hydroxyl, F, and other terminal groups.

**Figure 3:**
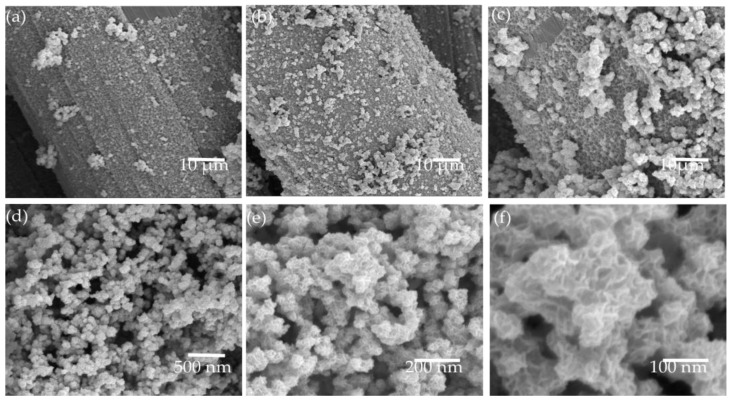
SEM images of synthesized MoSe_2_@Ti_3_C_2_T_x_/CC samples; (**a**) MoSe_2_@2 mgTi_3_C_2_T_x_/CC; (**b**) MoSe_2_@4 mgTi_3_C_2_T_x_/CC; (**c**) MoSe_2_@6 mgTi_3_C_2_T_x_/CC; and MoSe_2_ nanoflower on the samples of MoSe_2_@4 mgTi_3_C_2_T_x_/CC at (**d**) ×5000, (**e**) ×100,000, (**f**) ×200,000.

MoSe_2_@Ti_3_C_2_T_x_ was successfully synthesized on the Carbon Clothes with a facile one-step hydrothermal. During the preparation of the samples, the Ti_3_C_2_T_x_ played a key role, and SEM was used to observe the influence of the Ti_3_C_2_T_x_ on the surface morphology features of the synthesized samples. As shown in Figure 3a–c, with the increase of Ti_3_C_2_T_x_ content, more and more MoSe_2_ became attached to the carbon cloth substrate. This may be due to the intense attraction between terminal groups (-F, -OH, -O) on Ti_3_C_2_T_x_ and carbon fiber cloth substrate. However, the surface of MoSe_2_@4 Mg Ti_3_C_2_T_x_ had the most evenly distributed MoSe_2_ nanoflowers. A high-magnification scanning electron microscope was used to analyze its surface morphology further to study the MoSe_2_@4 Mg Ti_3_C_2_T_x_. High-magnification scanning microscope photographs of MoSe_2_ nanoflowers are shown in Figure 3d–f. The size distribution of a single nanoflower was between 30 and 60 nm.

**Figure 4:**
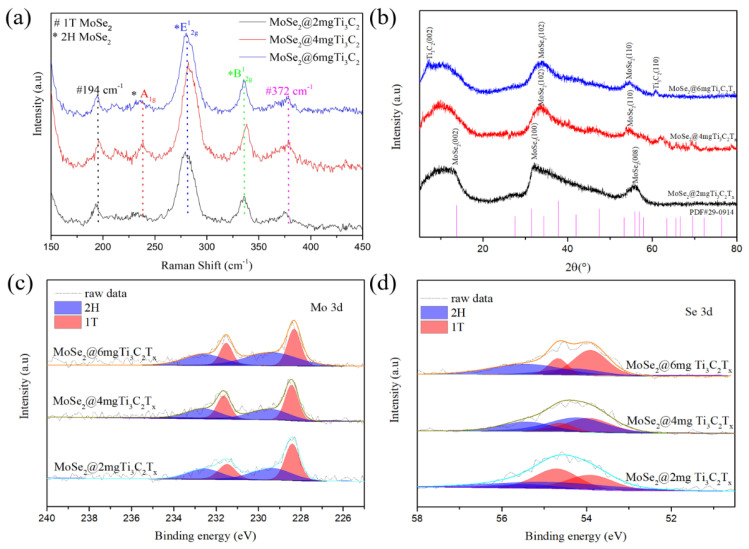
MoSe_2_ synthesized with different Ti_3_C_2_T_x_-added amounts; (**a**) Raman spectrometer shifts; (**b**) XRD pattern; (**c**) Mo 3d XPS spectra; and (**d**) Se 3d XPS spectra.

The Raman spectrum of all the samples show a characteristic peak at ≈285.4 cm^−1^. In accurate compliance to the in-plane (E^1^_2g_) mode, two other peaks at ≈239.4 cm^−1^ and ≈342.5 cm^−1^ on both sides of the main peak were found, (Figure 4a). Corresponding to A_1g_ and B^1^_2g_, the characteristic Raman manifested as 2H MoSe_2_ [34,35,36,37]. Furthermore, there are two inconspicuous peaks at ≈194 cm^−1^ and ≈376 cm^−1^, which correspond to the characteristic Raman mode of 1T MoSe_2_ [38,39]. However, with the increase of Ti_3_C_2_T_x_, there was no apparent shift in each peak position, and no characteristic peak of Ti_3_C_2_T_x_ was found in each sample; this may be due to the low amount of Ti_3_C_2_T_x_ or the adsorption of Ti_3_C_2_T_x_ on the carbon fiber cloth substrate. Raman spectra show that the MoSe_2_ we synthesized were 1T/2H multiple phases. To ascertain the existing phase composition and structural information of the as-synthesized samples XRD was carried out. The results are shown in Figure 4b; three prominent peaks were found which were located around 13.7°, 31.4° and 58°, respectively. Representing the (002), (110) and (008) crystal planes of MoSe_2_, due to the presence of surface structural defects, the (002) and (100) peaks of MoSe_2_ broaden in the XRD pattern [40]. Compared with the 2H-phase MoSe_2_ (JCPDS No 29–0914), standard XRD pattern means all the sample peaks of the (100) diffraction peaks shift right to a larger angle and the (002) crystal plane shifts left to a smaller angle, which is in good agreement with the result demonstrating the presence of 1T-phase MoSe_2_ in all synthesized samples [41,42]. With the increase in the amount of Ti_3_C_2_T_x_, a prominent peak corresponding to the (110) crystal plane of Ti_3_C_2_ was observed at 60.5° in MoSe_2_@4 mg. At Ti_3_C_2_T_x_, MoSe_2_@6 mg Ti_3_C_2_T_x_, another peak was observed near 7.3° and in MoSe_2_@6 mg Ti_3_C_2_T_x_, corresponding to the (002) crystal plane of Ti_3_C_2_. Therefore, we reached the preliminary conclusion that Ti_3_C_2_T_x_ did not decompose or participate in the reaction in the MoSe_2_@Ti_3_C_2_T_x_ synthesis. Next, XPS survey spectra illustrate that C, Ti, Mo, and Se elements are uniformly distributed in all samples. Next, the XPS spectra of MoSe_2_@Ti_3_C_2_T_x_ were further investigated for the presence of the 1T phase in the samples. Figure 4c shows the XPS spectrum in the Mo region. Two peaks at 54.7 and 53.7 eV correspond to 1T-phase MoSe_2_; double peaks at 228.3 and 231.4 eV correspond to Mo within the 1T phase, whereas 55.2 and 54.3 eV are assigned to the 2H phase MoSe_2_. The other pair at 229.3 eV and 232.5 eV can be assigned to the 3d_5/2_ and 3d_3/2_ orbitals of Mo within the 2H phase [43,44]. However, the 1T and 2H contents of each sample were different. The 1T-phase content in MoSe_2_@2 mg Ti_3_C_2_T_x_ was 64.4%; for MoSe_2_@4 mg Ti_3_C_2_T_x_, it was 66.9%; and the 1T-phase content in MoSe_2_@6 mg Ti_3_C_2_T_x_ was 54.58%. However, according to the previous report, temperature plays an important role in the formation of 1T MoSe_2_ [45]. Theoretically, there should be little difference in the 1T MoSe_2_ content of the sample. However, the addition of Ti_3_C_2_T_x_ may have affected the synthesis of 1T-phase MoSe_2_, but the terminal groups of Ti_3_C_2_T_x_ may prevent the formation of MoSe_2_. All the XPS results verified that 1T and 2H phases coexist in the samples we synthesized.

**Figure 5:**
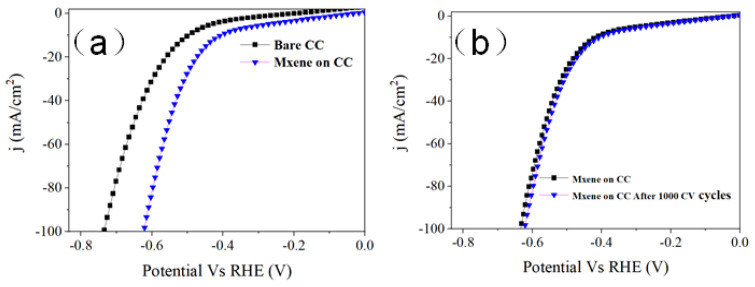
(**a**) LSV of Bare CC and MXene on CC; (**b**) LSV of MXene on CC and MXene on CC after 1000 CV cycles.

**Figure 6:**
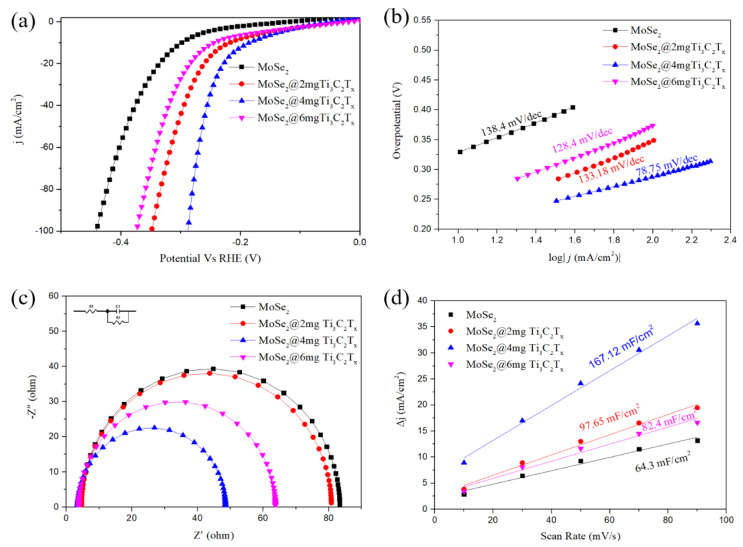
Electrochemical properties of MoSe_2_@ Ti_3_C_2_T_x_ synthesized with diverse amount of Ti_3_C_2_T_x_. (**a**) Polarization curves after IR calibration; (**b**) corresponding Tafel plots of the samples stemming from (**a**); (**c**) Nyquist plots of the samples; and (**d**) C_dl_ current at different scan rates.

We first tested the LSV curves for bare CC and MXene on CC, as shown in Figure 5. Dropping a small amount of MXene onto the carbon cloth can significantly improve the catalytic performance of the carbon cloth by about 2 mg. This may be due to the good conductivity of MXene. In addition, to determine the stability of MXene on the carbon cloth, we used a similar method in the paper 1000 CV cycles, and the performance of the sample did not change significantly. This may be due to the strong adsorption ability of MXene as a nanosheet and CC substrate, which did not considerably detach during the catalytic process. The HER electrochemical catalytic properties of MoSe_2_ obtained with a diverse mass of Ti_3_C_2_T_x_ were investigated in Figure 6a. According to XPS, the polarization curves after IR calibration are our work’s best value when the mass of Ti_3_C_2_T_x_ was 4 mg. The overpotential of η@10 mA/cm^2^ was 184. 4 mV. Still, with the increase of the Ti_3_C_2_T_x_, the overpotential of @6 mg Ti_3_C_2_T_x_ drops to 244.8 mV. This is due to its low 1T-phase content. However, compared with the sample without Ti_3_C_2_T_x_, using different masses of Ti_3_C_2_T_x_ suggests that the existence of Ti_3_C_2_T_x_ plays an important role in the majorization of HER electrocatalytic performance. The Tafel slope reflecting chemical reactivity in the HER further confirms the above results. Derived from their corresponding polarization curves, the samples prepared under different conditions are displayed in Figure 6b. The Tafel slope significantly decreased (79 mV dec^−1^), indicating a faster HER process in the sample. To understand the difference in the HER catalytic performance of the samples we synthesized varied Ti_3_C_2_T_x_ amounts in the Heyrovsky reaction rate-resolving stage. Furthermore, we analyzed CV measurement results to evaluate the effective electrochemical activity surface area (ECSA) by measuring *C*_dl_ stemming.

Samples with different component structures’ capacitive current and scan rates are presented in Figure 6c. MoSe_2_@4 mg-Ti_3_C_2_T_x_ also manifested larger *C*_dl_ (167.12 mF cm^−2^). Because of the existence of surface structural defects, the composite phase structure exposes more active site, which improves the performance of electrocatalytic HER. EIS tests were also conducted further to understand the electrochemical behavior in the HER process. Figure 6d presents the Nyquist plots of tested samples, displaying stable-system semicircle features. The charge-transfer resistance (R_ct_) can be obtained by calculating and fitting the Nyquist plot of Figure 6c. The R_ct_ values MoSe_2_@4 mg-Ti_3_C_2_T_x_ (23 Ω), MoSe_2_@2 mg-Ti_3_C_2_T_x_ (39 Ω), MoSe_2_@6 mg-Ti_3_C_2_T_x_ (30 Ω), and MoSe_2_ (40 Ω) results as mentioned above expound that the addition of Ti_3_C_2_T_x_ is critical to enhancing HER catalytic activity. From work obtained samples, MoSe_2_@4 mgTi_3_C_2_T_x_ that exhibit the best HER electrocatalytic performance.

**Figure 7:**
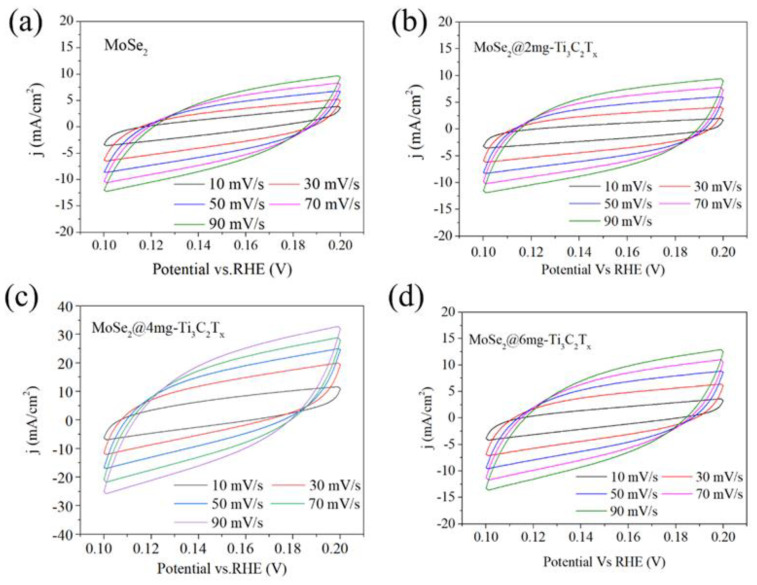
CV scanning curve in the scanning range of 0.1 V–0.2 V for different samples of (**a**) MoSe_2_, (**b**) MoSe_2_@2 mg-Ti_3_C_2_T_x_, (**c**) MoSe_2_@4 mg-Ti_3_C_2_T_x_, and (**d**) MoSe_2_@6 mg-Ti_3_C_2_T_x_.

The double plate capacitance extracted in the nonfaradaic-zone CV cycle is proportional to the electrochemical activation area of the catalytic electrode. Figure 7 shows the cyclic voltammetry curve of the sample for different MoSe_2_@Ti_3_C_2_T_x_. The sample was subjected to cyclic voltammetry testing. The test solution was consistent with the previous electrochemical workstation and still used a 0.5 mol/L H_2_SO_4_ solution. The scanning rates were 10, 30, 50, 70, and 90 mV/s. In addition, to ensure the stability of the sample, the last one was selected for 100 cycles at each rate and plotted. As shown in Figure 7a, the MoSe_2_ sample electrode without adding Ti_3_C_2_T_x_ showed the smallest capacitive behavior, followed by the electrode sample with the added 2 mg Ti_3_C_2_T_x_. As the content of Ti_3_C_2_T_x_ increases, the area of the CV curve gradually increases. The sample with a dosage of 4 mg Ti_3_C_2_T_x_ has the largest CV area. When the dosage of Ti_3_C_2_T_x_ is further increased to 6 mg, the geometric area of the CV curve shows a significant reduction trend. This indicates that the doping amount of Ti_3_C_2_T_x_ has an optimal value. When the doping amount of Ti_3_C_2_T_x_ reaches an optimal value, its catalytic activity will exceed the contribution of the carbon cloth matrix, becoming the dominant role instead. However, its performance will gradually decline after exceeding a specific optimization interval. Based on the previous XPS characterization results, this is due to the addition of a large amount of Ti_3_C_2_T_x_, which is affected by the adsorption of terminal groups -F, -OH, etc., on the surface of Ti_3_C_2_T_x_. The solution’s environment during the preparation of 1T phase is disrupted, thereby affecting the generation of 1T-phase MoSe_2_, and reducing the content of 1T phase in the sample. In addition, due to the reduction of MoSe_2_ content in the 1T phase, the number of hydrogen evolution catalytic active sites introduced by the disordered arrangement of atoms at the interface of 1T and 2H phase in the sample due to different structures, will also be reduced. This further affects its catalytic activity and reduces the CV area at different scanning rates.

We investigated the catalytic performance of Ti_3_C_2_T_x_ during the in situ growth of MoSe_2_ electrodes on carbon cloth. The results showed that adding Ti_3_C_2_T_x_ optimized the electrocatalytic hydrogen evolution performance of MoSe_2_ electrodes to varying degrees. Based on characterization methods, it is speculated that adding Ti_3_C_2_T_x_ changed the in situ growth process of MoSe_2_ on carbon cloth. Due to the introduction of active groups, such as F during the preparation of Ti_3_C_2_T_x_, it is more inclined to adhere to the carbon cloth. On the other hand, as a two-dimensional material, it is easy to synthesize heterostructures with MoSe_2_ based on the interlayer van der Waals force. In addition, its natural good conductivity can act as a good electronic channel between MoSe_2_ and the carbon cloth. Therefore, MoSe_2_ not only exposes more electrocatalytic active sites but also greatly improves the adhesion between the catalyst and the carbon cloth, so it can effectively optimize the electrocatalytic performance of MoSe_2_. Based on the successful preparation of 1T/2H impure-phase MoSe_2_, we investigated the effect of Ti_3_C_2_T_x_ addition on the electrocatalytic performance of MoSe_2_. For samples with a low amount of Ti_3_C_2_T_x_ addition, Ti_3_C_2_T_x_ cannot perform its expected conductivity, and the optimization of MoSe_2_ electrocatalytic performance is limited. However, when more Ti_3_C_2_T_x_ is added, the terminal groups adsorbed on the surface of Ti_3_C_2_T_x_ will affect the formation of 1T-phase MoSe_2_. With the increase of Ti_3_C_2_T_x_ addition, the sample with 4 mg Ti_3_C_2_T_x_ exhibited good electrocatalytic hydrogen evolution performance.

As shown in Table 1, the enhanced catalytic performance is attributed to a larger density of exposed catalytically active edge sites and a higher electrical conductivity. Other studies on improved catalytic activity of MoSe_2_ have shown that the structure of the high-content 1T phase with defects is instrumental in enhancing HER performance [46].

**Figure 8:**
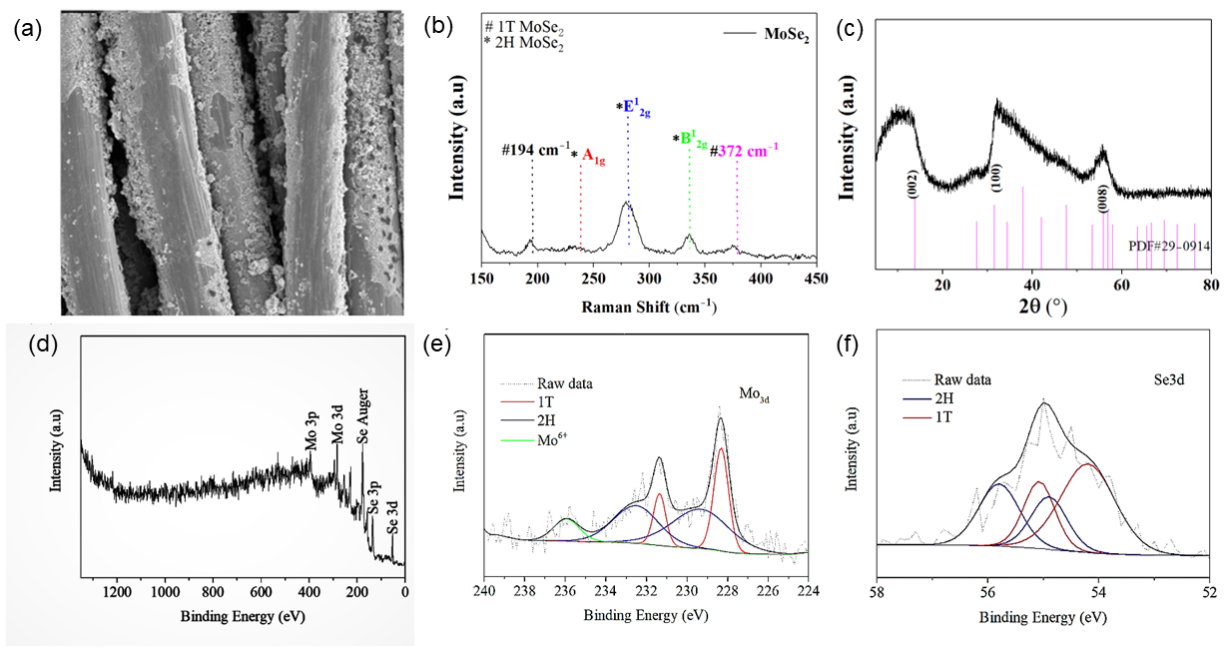
Characterization of MoSe2@2 mg Ti_3_C_2_T_x_ after CV test samples of (**a**) SEM, (**b**) Raman spectrum, (**c**) XRD spectrum, (**d**) XPS full spectrum, (**e**) XPS Mo 3d peak, and (**f**) XPS Se 3d peak.

To study the stability of the sample, we conducted experiments on the stability of the samples MoSe_2_@Ti_3_C_2_T_x_. After the CV test, the following experiments were carried out: the SEM of samples shown in Figure 8a, and the sample Raman spectroscopy shown in Figure 8b. There were no shifts for the Raman peak of 1T and 2H MoSe_2_ after the test, and the main crystal planes remain the same as before the CV test as shown in Figure 8c, and the half-peak width did not change significantly, indicating that the test did not significantly change its crystal structure. We used XPS to illustrate whether MoSe_2_ transitioned from 1T to 2H after the test, since phase 2H is a thermodynamically stable phase. As shown in the figure, the chemical elements and valence states that the full spectrum of XPS did not change significantly, and in spectroscopy, we used the same method as in the paper to split the Mo 3d and Se 3d peaks, respectively. However, the phase content in MoSe_2_@2 mg Ti_3_C_2_T_x_ after the CV test decreased from 64.4% to 63.3%. This reduction is tolerable since the samples were dried before testing.

**Figure 9:**
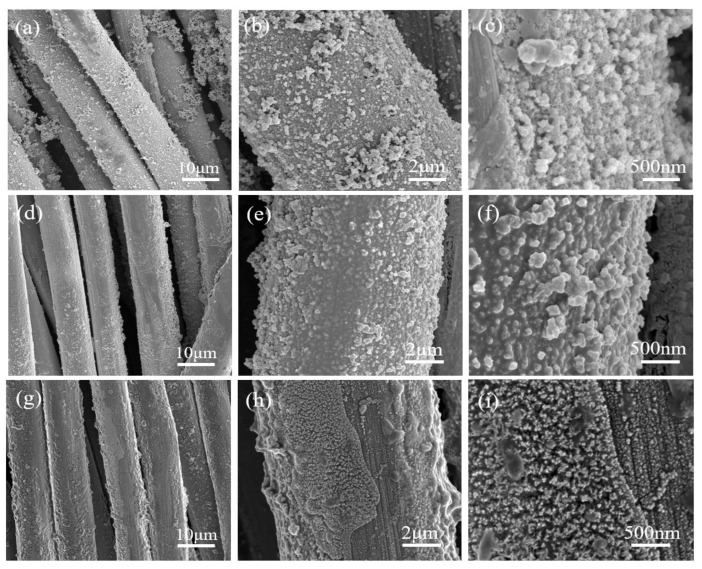
(**a**–**c**) Carbon cloth in situ growth MoSe_2_@4 mg Ti_3_C_2_T_x_ initial surface morphology of sample (**d**–**f**) after 500 Cycles, and microscopic morphology of the sample at different magnification times, and (**g**–**i**) after 1000 cycles, and microscopic morphology of the sample at different magnification times.

In addition, the surface morphology of the samples after different cycles was observed. Figure 9a–c show the initial morphology of the sample without cycling. It can be seen that a large number of MoSe_2_ clusters uniformly adhere to the surface of the sample, and there is also a small amount of MoSe_2_ powder attached to the surface of the carbon cloth fiber. Still the contribution to hydrogen evolution performance is extremely limited due to the small contact area with the carbon cloth. A clear tissue network can be seen with significant adhesion to the substrate at high magnification. Figure 9d–f shows the surface morphology of the sample after 500 cycles, and MoSe_2_ powder on the surface of the carbon cloth has obviously fallen off. Still, the MoSe_2_ clusters adsorbed on the surface have not obviously fallen off. Still the cluster morphology has changed greatly, the MoSe_2_ on the surface has changed from loose nanoflower clusters to “chocolate” like whole blocks attached to the surface of the carbon cloth, and small MoSe_2_ particles can still be seen at local magnification. The particles are closely bound to the carbon cloth substrate. In Figure 9g–i, a large number of MoSe_2_ nanoparticles are still uniformly adhered to the surface of the sample after 1000 cycles. However, the difference is that the particle size is significantly reduced under local magnification, with a small amount of detachment locally. Overall, the majority of MoSe_2_ has a relatively tight binding force. It is worth noting that Nafion and other reagents were not used to promote the adhesion of MoSe_2_ on the substrate during this experimental testing process. The small amount of MoSe_2_ attached to the surface of the carbon cloth itself has a limited contribution to the catalytic performance of the catalyst due to its small interface with the carbon cloth, and its detachment will not significantly impact the catalyst performance.

## 4. Conclusions

In this work, the two-dimensional Ti_3_C_2_T_x_ was added to form the heterostructure with 1T/2H MoSe_2_ to improve the contact with the conductive carbon fiber cloth substrate. In addition, Ti_3_C_2_T_x_ itself, as a good conductor, can provide fast channels and more active sites for electron transfer during electrochemical reactions. According to the control, the addition of a mass of Ti_3_C_2_T_x_ to form proper MoSe_2_/Ti_3_C_2_T_x_ heterostructures with low overpotentials (−185.29 mV) and small Tafel slopes (79 mV dec^−1^) are achieved with the obtained MoSe_2_@4 mg-Ti_3_C_2_T_x_, Moreover, phase engineering synergistic effects and remaking heterostructures using MoSe_2_ with other materials can also be used for MoSe_2_ future development in the field of electrochemical catalysis. However, how the terminal groups of Ti_3_C_2_T_x_ influence the formation of the 1T MoSe_2_ is still not clear. More comparative experiments need to be carried out with further Ti_3_C_2_T_x_ addition.

## Figures and Tables

**Table 1:** Electrochemical performance (j = 10 mA cm^−2^) comparison of different MoSe_2_-based composite samples.

Sample	Overpotential (V vs. RHE)	Tafel Slope (mV dec^−1^)	References
2H MoSe_2_	0.300	82	[47]
MoSe_2_-amorphous CNT	0.254	49	[48]
C@MoSe_2_	0.270	72	[49]
Graphene-carbon nanotube aerogel-MoSe_2_ hybrid	0.228	68	[50]
N-doped RGO/MoSe_2_ composites	0.229	78	[51]
MoSe_2_/Ti_3_C_2_T_x_	0.185	79	This work

## Data Availability

Not applicable.
